# Influence of weaning methods on the diaphragm after mechanical ventilation in a rat model

**DOI:** 10.1186/s12890-016-0285-2

**Published:** 2016-08-24

**Authors:** Christian S. Bruells, Thomas Breuer, Karen Maes, Ingmar Bergs, Christian Bleilevens, Gernot Marx, Joachim Weis, Ghislaine Gayan-Ramirez, Rolf Rossaint

**Affiliations:** 1 Department of Intensive and Intermediate Care, University Hospital of the RWTH Aachen, Pauwelsstr. 30, 52074 Aachen, Germany; 2 Department of Anaesthesiology, University Hospital of the RWTH Aachen, Pauwelsstr. 30, 52074 Aachen, Germany; 3 Laboratory of Pneumology, Katholieke Universiteit Leuven, Leuven, Belgium; 4 Institute of Neuropathology, University Hospital of the RWTH Aachen, Aachen, Germany

**Keywords:** Mechanical ventilation, Pressure support ventilation, Spontaneous breathing trial, Ventilator-induced diaphragmatic dysfunction, Weaning

## Abstract

**Background:**

Mechanical ventilation (MV) is associated with diaphragm weakness, a phenomenon termed ventilator-induced diaphragmatic dysfunction. Weaning should balance diaphragmatic loading as well as prevention of overload after MV. The weaning methods pressure support ventilation (PSV) and spontaneous breathing trials (SBT) lead to gradual or intermittent reloading of a weak diaphragm, respectively. This study investigated which weaning method allows more efficient restoration of diaphragm homeostasis.

**Methods:**

Rats (*n* = 8 per group) received 12 h of MV followed by either 12 h of pressure support ventilation (PSV) or intermittent spontaneous breathing trials (SBT) and were compared to rats euthanized after 12 h MV (CMV) and to acutely euthanized rats (CON). Force generation, activity of calpain-1 and caspase-3, oxidative stress, and markers of protein synthesis (phosphorylated AKT to total AKT) were measured in the diaphragm.

**Results:**

Reduction of diaphragmatic force caused by CMV compared to CON was worsened with PSV and SBT (both *p* < 0.05 vs. CON and CMV). Both PSV and SBT reversed oxidative stress and calpain-1 activation caused by CMV. Reduced pAKT/AKT was observed after CMV and both weaning procedures.

**Conclusions:**

MV resulted in a loss of diaphragmatic contractility, which was aggravated in SBT and PSV despite reversal of oxidative stress and proteolysis.

**Electronic supplementary material:**

The online version of this article (doi:10.1186/s12890-016-0285-2) contains supplementary material, which is available to authorized users.

## Background

Mechanical ventilation (MV) is a life preserving technique in all patients with acute respiratory failure or need for deep sedation. During this period the diaphragm remains inactive and is hampered after several hours of inactivity, referred to as ventilator-induced diaphragmatic dysfunction (VIDD) [1, 2]. Generation of mitochondrial reactive oxygen species (ROS) has been revealed to play an essential role [3, 4], and is pre-requisite for further degradation by proteases [5, 6], autophagy [2], and the proteasome system [7]. In addition, protein synthesis is hampered via the protein kinase B (AKT) pathway [1, 8].

Weaning failure in patients remains a serious concern and VIDD is suspected to play a key role in this process. The question how fast the diaphragm recovers after occurrence of VIDD remains controversial [9–11]. These studies suggest that diaphragm function recovery seems to dependent on load and mutual prevention of overload.

These data further support the idea that balancing between reloading and prevention of overload is the central issue in weaning patients from the ventilator [12]. Actually, two weaning methods are recommended in recent guidelines, spontaneous breathing trial (SBT) allowing intermittent but full diaphragm reloading or pressure support ventilation (PSV) [13], causing gradual reloading. It is, however, not known, which of these recommended weaning modes would lead to the most efficient diaphragm regaining in terms of cellular homeostasis and force.

We hypothesized that diaphragm reloading with PSV or hourly SBT after 12 h of MV would lead to a fast and complete recovery of diaphragm contractile force and cellular homeostasis.

## Methods

The study protocol was approved by the ethics committee (Landesamt für Natur, Umwelt und Verbraucherschutz Nordrhein-Westfalen, Germany, Permit number: AZ 84–02.04.2011.A277).

### Design

Healthy, 450 g, male Sprague–Dawley rats were tracheotomised and submitted to mechanical ventilation for 12 h under pentobarbital anaesthesia (Babylog 3000, Draeger, Luebeck, Germany). After this intervention, animals were separated into 3 groups (*n* = 8 each). The first group was euthanized after these initial 12 h (CMV). The second group breathed spontaneously under pressure support ventilation (PSV) for 12 h and the third group underwent an intermittent spontaneous breathing trial (SBT), consisting of 5 min spontaneous breathing each hour followed by 55 min of controlled mandatory ventilation. An acutely anaesthetized group served as control (CON; *n* = 8). In respect of animal care and the obvious time dependence of VIDD as shown in many studies, we decided not to examine 24 h of CMV under pentobarbital anaesthesia as an additional control [14, 15]. No muscle relaxant was applied in any group.

### Experimental procedure

Anaesthesia was initiated by intraperitoneal injection of 60 mg Pentobarbital/kg of bodyweight and sustained by i.v. administration. During weaning, pentobarbital infusion was lowered to allow spontaneous breathing but keep an appropriate level of sedation present to protect the animals from stress.

The interventional groups were tracheotomised and a pressure-controlled ventilation was applied using a respiratory rate of 60 bpm, a positive end-expiratory pressure of 3 cmH_2_O, a tidal volume of 6–7 ml/kg and I:E ratio of 1:1 to maintain a PaO_2_ of 70–100 mmHg.

**Fig. 1:**
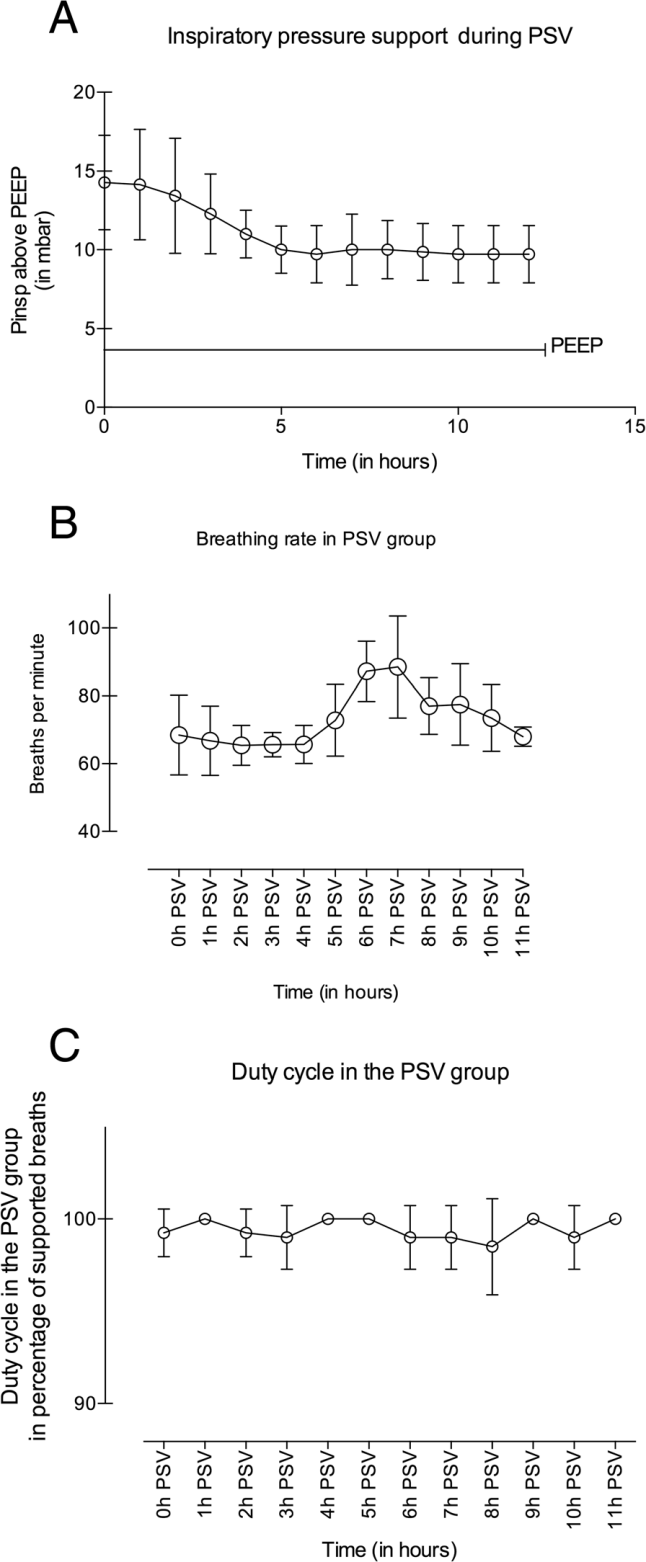
Breathing rates, duty cycles and inspiratory pressure support in the PSV group. Values are expressed as means ± SD

All animals were continuously monitored [11]. A catheter was inserted into the oesophagus to measure oesophageal pressure and detect insufficient diaphragm / ventilator interaction. Every 3 h arterial blood samples were taken for measurement of blood gases. After 12 h of MV, animals of the SBT group were removed from the ventilator to breathe spontaneously every hour for 5 min [8] during the 12 h study period. After 12 h of MV, animals in the PSV group were ventilated with pressure support ventilation. The trigger level, which is a relative number in the ventilator to be set between 1 and 10 in 0.1 increments, was set to allow an evident triggering of each breath without inducing an auto-trigger. The level was therefore raised from the lowest trigger level until no auto-trigger occurred anymore, detected as pressure drop in the oesophageal pressure. The inspiratory pressure was set at the same level as during CMV to uphold normocarbia. This level was reduced hourly by 1 cmH_2_O. The level was adapted and increased when the respiratory rate increased above 100 bpm or hypercarbia occurred despite sufficient respiratory rate. The minimum inspiratory pressure to be delivered was 5 cmH_2_O due to ventilator pre-settings (Fig. 1).

### Control group

In the CON group, the diaphragm was removed after tracheostomy and a short ventilation period (maximally 5 min) to prevent hypercarbia.

#### Functional and histological measurements

At the end of the experimental period, a segment of the costal diaphragm was removed and muscular force was assessed in vitro using our established protocol [11].

Another strip was embedded and frozen in liquid butane for histological assessment of muscle fibre dimensions as described before [16]. Fibre cross-sectional areas were examined using immunofluorescence microscopy (approximately 200 fibres per animal) and analysed using the software ImageJ (v1.46 k; National Institute of Health, Bethesda, MA) [17].

#### Analysis of diaphragmatic inflammation

To asses diaphragmatic leukocyte activity neutrophils, ED1+ macrophages, and ED2+ macrophages were analysed using our established immunohistochemistry protocol [9]. Cell densities were expressed as the number of labelled cells per cubic millimetre.

#### Biochemical measurements

### Western blotting

Total protein concentration was determined with the Bradford method [18]. Proteins were separated on a polyacrylamide gel (6 for proteolysis markers, 12 for protein oxidation and 10 % for others) and transferred onto a polyvinyldifluoride membrane. Ponceau S staining was used to ensure equal loading and proper transfer of the proteins. Blots were incubated overnight at 4 °C with appropriate primary antibody and subsequently with the suitable secondary antibody. Proteins were visualized with chemiluminescent Peroxidase Substrate (Sigma-Aldrich, Bornem, Belgium) and analysed with the software package (Bio 1D) of the imaging system (Photo print, Vilber, France).

### Oxidative stress

Total protein carbonyl levels in the diaphragm were measured using the Oxyblot Protein Oxidation Detection Kit (Chemicon, Temecula, CA) following manufacturer’s instructions. The oxidative index was defined as ratio between densitometric values of the oxidized proteins and the Ponceau S stained bands [19].

Diaphragmatic 4-hydroxynonenal (4HNE) was used as an index of lipid peroxidation. Polyclonal anti-4-HNE antibody (MAB32495, R&D Systems, Abingdon, UK) was used as primary antibody and a polyclonal rabbit anti-mouse (P0260, Dako, Heverlee, Belgium) as secondary. Data were expressed as a ratio between densitometric values of 4-HNE and Ponceau S.

### Protein synthesis

To assess the mTOR pathway, the phosphorylated AKT protein was first detected using a rabbit polyclonal primary antibody against total AKT (Cell Signaling Technology, Danvers, MA), and HRP-conjugated goat anti-rabbit secondary antibody (Cell Signaling Technology, Danvers, MA). After detection, the blot was stripped for detection of total AKT (Cell Signaling Technology, Danvers, MA). The data were expressed as the ratio between band intensity of phosphorylated AKT (pAKT) to total AKT.

### Proteolysis

Calpain and caspase-3 activities were measured indirectly by assessing the breakdown products of α-II spectrin (FG6090–0100, Enzo Life Sciences, Antwerp, Belgium), a specific substrate of calpain and caspase-3. The cleavage product of intact α-II spectrin by calpain gives band at 150 kDa and at 120 kDa when cleaved by caspase-3 while intact α-II spectrin is detected at 260 kDa when labelled using a secondary antibody (polyclonal rabbit anti-mouse P0260, Dako, Heverlee, Belgium). Calpain and caspase-3 activities are expressed as the ratio between the densitometric values of their breakdown products to the intact α-II spectrin [19].

#### Statistical analysis

All data were analysed in masked randomization. Population distribution was assessed with the Kolmogorov-Smirnov test. Normally distributed data were compared using one-way analysis of variance (ANOVA) and for the force frequency measurements using a two-way repeated-measures ANOVA. If the group effect was significant, a Tukey post-hoc test was used for pairwise comparisons between all groups. Otherwise a Kruskal-Wallis test followed by a Dunn’s post-hoc test, was used as indicated in the results section. Data are shown as means ± SD or median ± SEM, when appropriate. All statistical tests are two-tailed, significance was established at *p* < 0.05 (GraphPad Prism 6.0, La Jolla, CA).

## Results

### Physiological variables during the experiment

Arterial blood pressure was stable throughout the entire experimental period. Blood pressures were 124 ± 22 mmHg (CMV), 111 ± 13 mmHg (PSV) and 94 ± 12 mmHg (SBT) before sacrifice. Data of blood gases and pH did not differ between all groups and remained inside physiological ranges (Table 1, Additional file 1). The trigger setting in the PSV group allowed a nearly complete synchronisation between animal breaths and the pressure support. With decreasing pressure support, breathing frequency increased in the animals (Fig. 1).

**Table 1:** Blood gases of the four groups at the end of the experiment. Values are means ± SD

	CON	CMV	PSV	SBT
paO_2_ (mmHg)	91 ± 21	112 ± 19	104 ± 13	111 ± 16
paCO_2_ (mmHg)	38 ± 9	37 ± 8	37 ± 13	35 ± 9
pH	7.41 ± 0.05	7.47 ± 0.07	7.38 ± 0.07	7.45 ± 0.08

### Diaphragm function

**Fig. 2:**
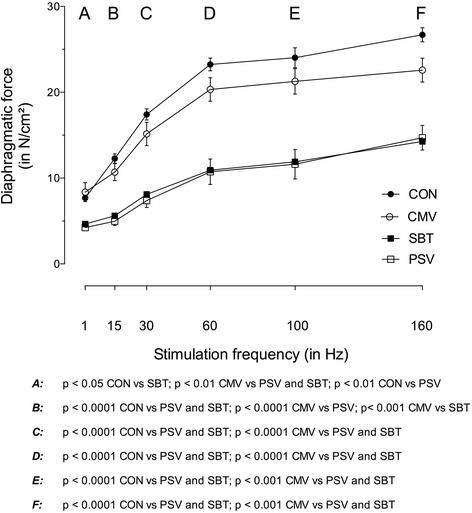
Force frequency relationship of the diaphragm in vitro. The weaning groups show a significant decrease in contractile force at all stimulation frequencies compared to CON and CMV. **a** indicates *p* < 0.05 SBT vs. CON, *p* < 0.01 PSV vs. CON and PSV/SBT vs CMV; **b** indicates *p* < 0.0001 PSV/SBT vs. CON, PSV vs. CMV and *p* < 0.001 SBT vs. CMV; **c** *and* **d** indicate *p* < 0.0001 PSV/SBT vs. CON and CMV; **e** *and* **f** indicate *p* < 0.0001 PSV/SBT vs. CON and *p* < 0.001 PSV/SBT vs. CMV. Values are shown as means and SD

The diaphragm force frequency curve was shifted downwards significantly at all stimulation frequencies in the PSV and SBT groups compared to CON and CMV (Fig. 2). The interaction between frequency and group was significant (*p* < 0.0001), indicating significant differences between groups at all stimulation frequencies (Fig. 2). The loss of diaphragm force in the PSV and SBT groups compared to controls was similar (e.g. 160 Hz: 45 and 47 %, respectively).

### Diaphragm histology

Histological planimetry of diaphragmatic fibre dimensions did not show any significant differences in fibre cross sectional areas for all groups compared to CON (Table 2).

**Table 2:** Diaphragmatic cross sectional areas (in μm^2^) of type I, IIA and IIx/b fibres for CON (*n* = 8), CMV (*n* = 8), PSV (*n* = 7) and SBT (*n* = 7). Values are means ± S

Type I	Type IIa	Type IIx/b
CON		
1838 ± 223	1886 ± 273	4445 ± 971
PSV		
1693 ± 276	1847 ± 249	3978 ± 1264
CMV		
1838 ± 294	1823 ± 454	4000 ± 835
SBT		
1788 ± 320	1830 ± 389	4466 ± 1172

#### Diaphragmatic inflammation

**Fig. 3:**
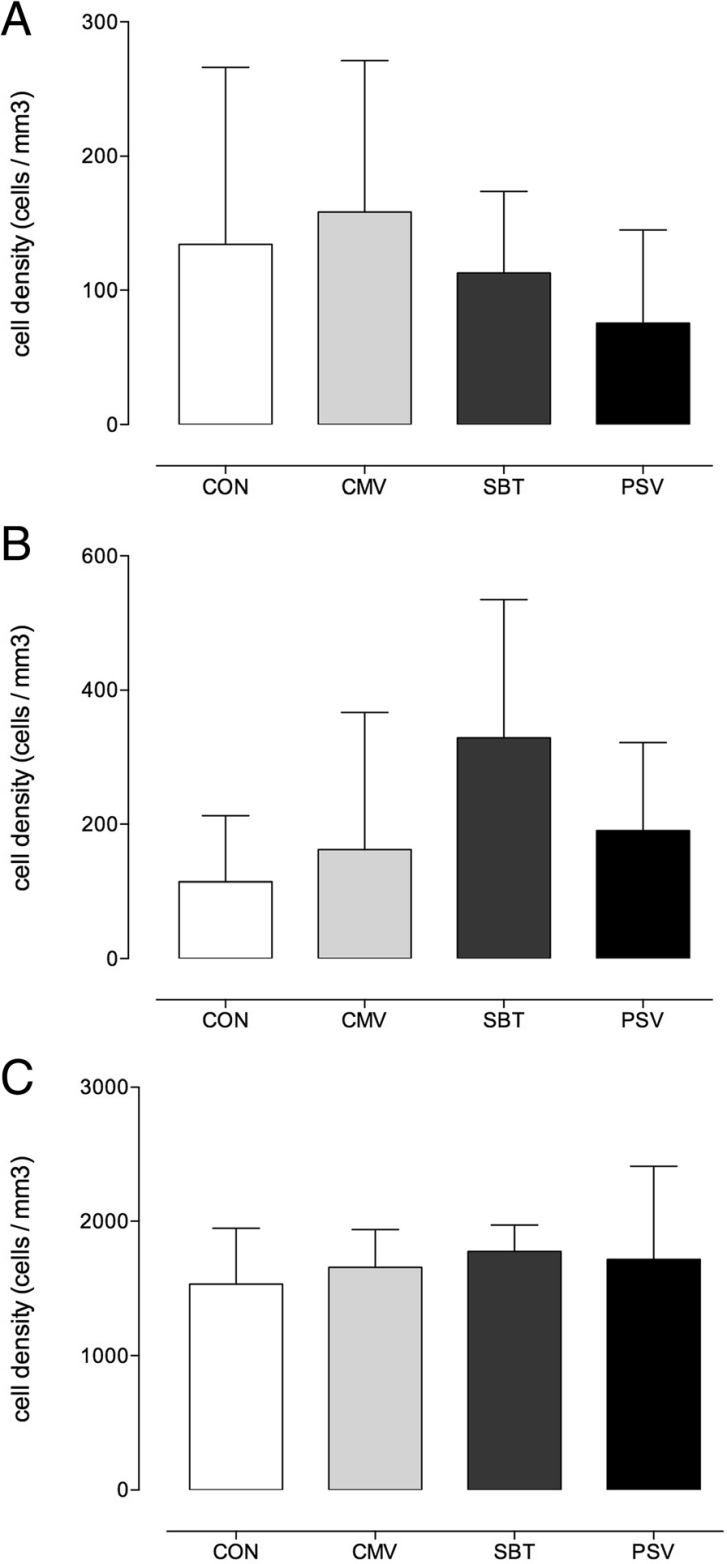
Diaphragmatic leukocyte activity measured as neutrophils panel **a**, ED1+ macrophages panel **b**, and ED2+ macrophages panel **c**. Cell densities were expressed as the number of labelled cells per cubic millimetre. Values are means ± SD

Neutrophilic and macrophagic invasion were unaltered as no significant differences in the quantity of neutrophils, ED1+, and ED2+ macrophages between all groups were detected (Fig. 3).

### Oxidative stress

**Fig. 4:**
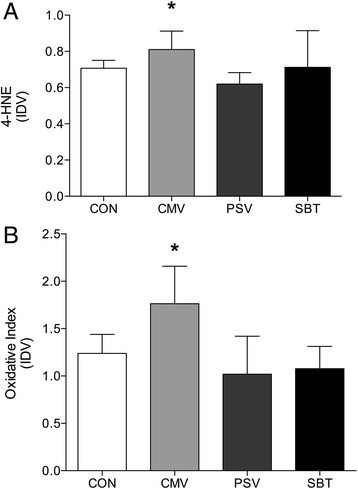
Levels of lipid peroxidation expressed as a ratio between densitometric values of 4-HNE and Ponceau S (panel **a**) and oxidized proteins as a ratio between densitometric values of the oxidized proteins and Ponceau S (panel **b**) measured in the diaphragm after only 12 h of CMV (CMV) or 12 h CMV and additional 12 h of weaning (PSV, SBT) compared to CON. * *p* < 0.05 CMV vs. PSV (panel **a**) and CMV vs. other groups (panel **b**). Values are means ± SD. IDV: Integrated Density Value

Diaphragmatic 4-HNE levels were significantly increased after CMV compared to PSV (+38 %, *p* = 0.034). There were no differences in 4 HNE levels between PSV, SBT, and CON (Fig. 4, panel a). Protein oxidation was significantly increased by 42 % in the CMV compared to others (vs. PSV *p* = 0.004; vs. SBT *p* = 0.007 and vs. CON *p* = 0.047) (Fig. 4, panel b) but was similar to CON levels in the PSV and SBT groups.

### Markers of protein synthesis

**Fig. 5:**
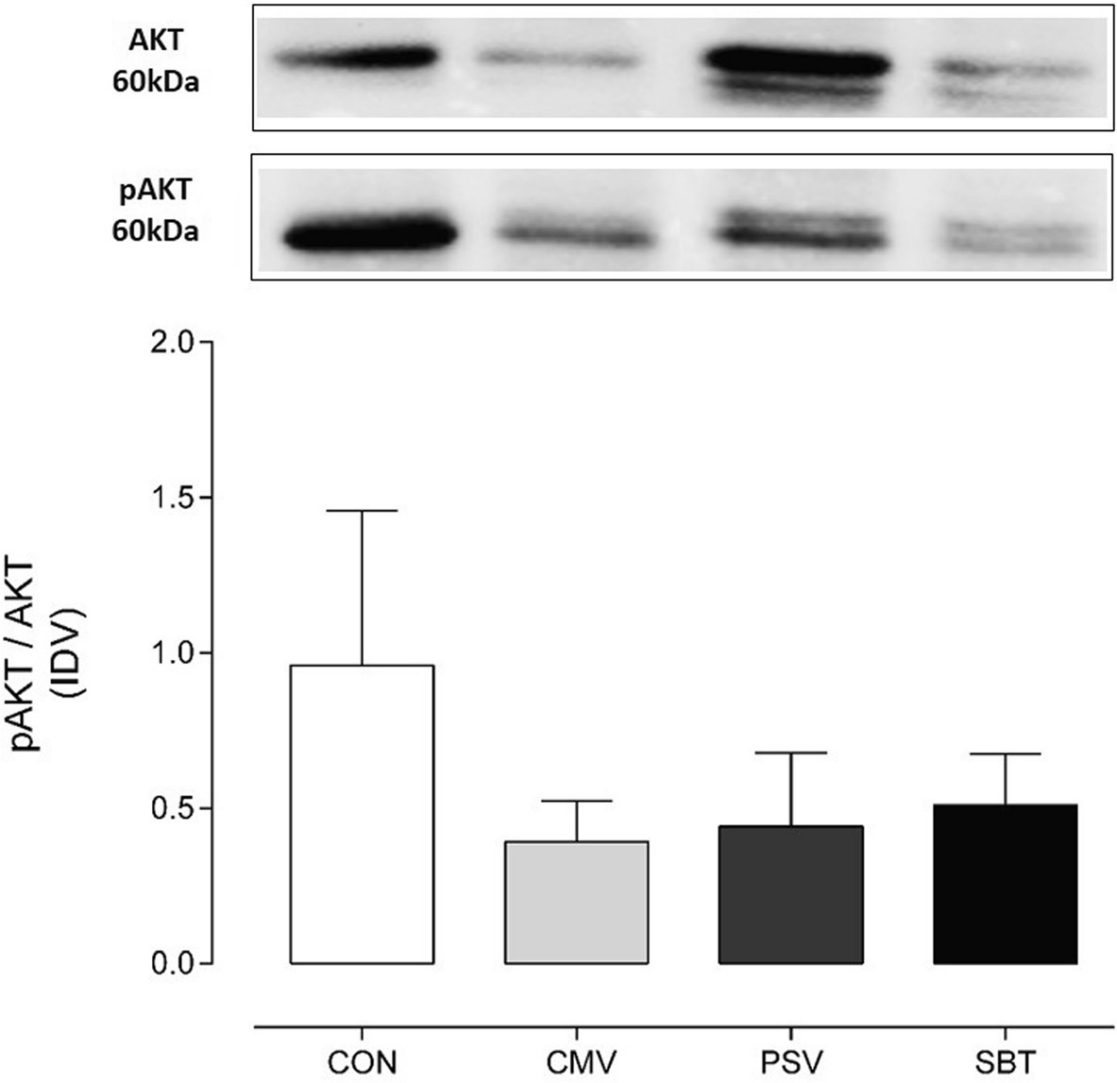
Diaphragm levels of activated AKT expressed as the ratio of pAKT/AKT in the different groups. Values are mean ± SD

Compared to CON, pAKT/AKT ratio was decreased to a similar extent after CMV (−59, *p* = 0.06), SBT (−46, *p* = 0.06), and PSV (−54 %, *p* = 0.1) but this reduction failed to reach statistical significance (Fig. 5).

### Proteolysis

**Fig. 6:**
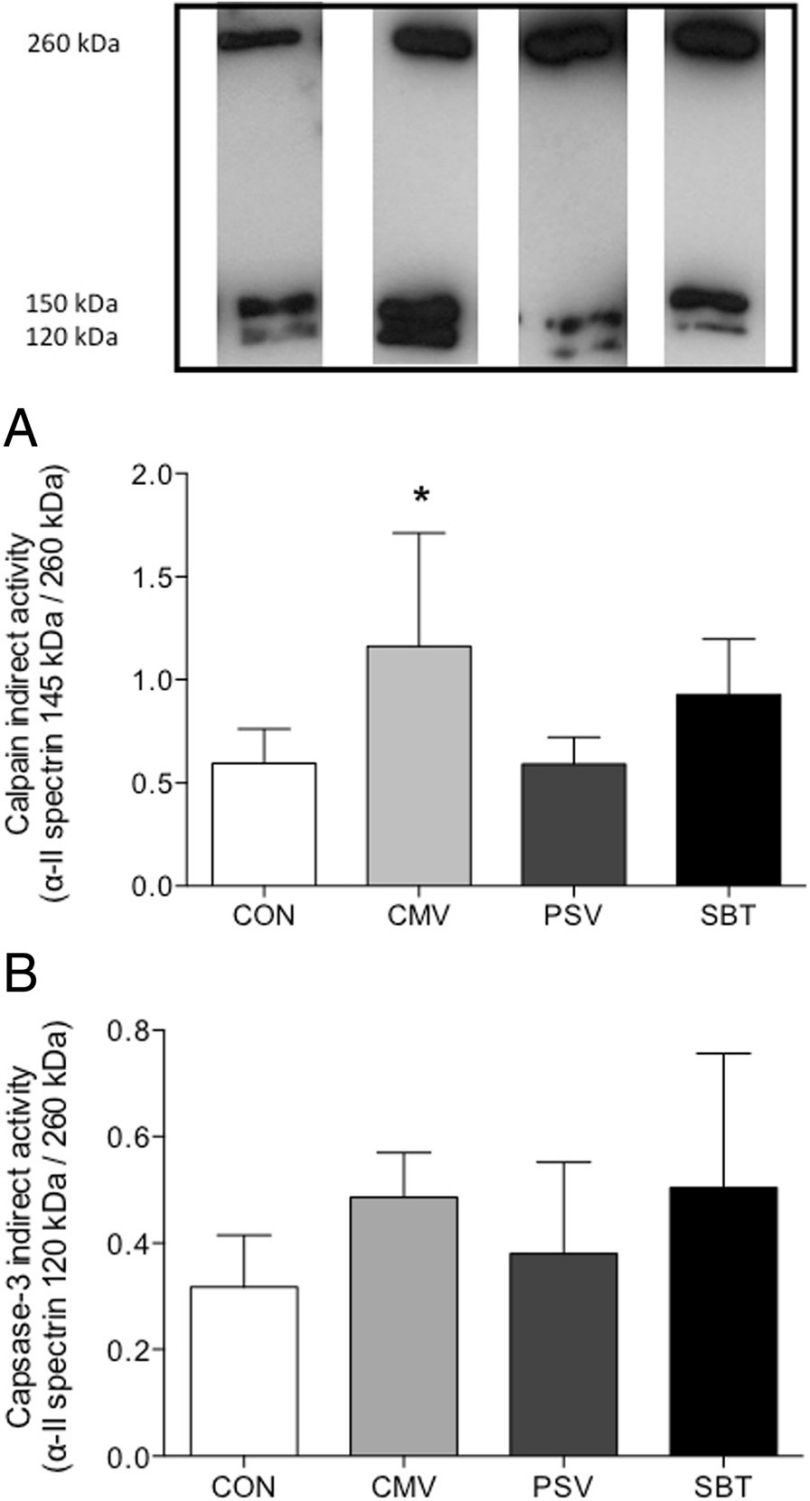
Indirect assessment of calpain activity (panel **a**) and caspase-3 activity (panel **b**) in the different groups. 120 kDa/260 kDa and 150 kDa/260 kDa represent the ratio of breakdown product to intact αII-spectrin induced by calpain and caspase-3, respectively. Values are mean ± SD. *p < 0.05 vs. CON and PSV. Values are expressed as means ± SD

Calpain activity was significantly elevated in CMV (+95 %, *p* = 0.02) compared to CON and PSV while it was similar to CON levels in the PSV and SBT groups (Fig. 6, panel a). For caspase-3 activity no significant differences were found between the groups (Fig. 6, panel b).

## Discussion

### Overview over principle findings

This is to our knowledge the first study examining the impact of weaning methods PSV and SBT on diaphragm function and metabolism after the onset of VIDD. Our data show that both strategies further reduced diaphragm force despite the fact that they reversed oxidative stress and proteolysis caused by mechanical ventilation.

These findings are discussed in detail below.

### Weaning using SBT and PSV does not result in diaphragm function recovery

In the current study, neither SBT nor PSV applied as weaning strategies after 12 h of CMV were associated with diaphragm function recovery. In fact, each strategy led to a worsening of diaphragm function and intriguingly the magnitude of this attenuation was similar whether the diaphragm was gradually (PSV) or intermittently fully (SBT) reloaded. Our data are surprising in the context of previous studies, which demonstrated a rapid regaining in diaphragm force within 4–7 to 24 h after induction of VIDD, when the animals breathed completely independent from further assistance but with the risk of muscle overload and inflammatory damage [9, 11]. Importantly, in the PSV group the inspiratory pressure delivered by the ventilator was decreased, i.e. the diaphragm had to contract more forcefully; obviously, the increase of work was not sufficient to regain force.

Although 5 min of spontaneous breathing seem to protect, at least partially from a contractile deficit [8], it is obviously not enough to regain muscle force. Martin and colleagues proposed in patients an inspiratory muscle training of very low duty cycles (i.e. 40 breaths per day), resulting in a better patient inspiratory force [20]. From our data we cannot support these findings that a low duty cycle might be enough to regain muscle force.

Theoretically, this decline in force could be induced by overload; nevertheless the triggering was carefully set in the PSV mode and airway obstruction during the 5 min SBT phases were avoided.

### SBT and PSV reverse diaphragmatic oxidative stress

MV resulted in an increase in oxidized proteins and lipid peroxidation making sarcomeres more susceptible for the breakdown by the protease system [6]. This finding is in line with former publications from our groups [6, 11, 21], defining it as one major pathway of VIDD [22]. The reduction in oxidative damage during the weaning process might result in less cleavage of sarcomeric proteins.

Both weaning strategies were able to reverse the amount of oxidized proteins and lipid peroxidation but this did not result in diaphragmatic functional improvement. Muscle force depends on the number of myosin heads forming parallel cross bridges per half sarcomere, the steady state fraction of strongly bound cross bridges in the force generating state and the force generated by cross bridges [23]. Mitochondrial uncoupling may be reduced by activation of any kind as in our model and result in decreased oxidative damage of cellular structures [23], but this had no impact on force generation.

### Weaning reverses the activation of the calpain system

Mechanical ventilation induced an elevation of calpain activity compared to CON in line with former publications from our group and others [24, 25]. The increase in calpain activity was significantly down regulated after weaning by PSV compared to CMV, but not in SBT group. Previous studies examining diaphragmatic recovery after MV reported various effects on these proteases after reloading [5, 26, 27]. Thus, full diaphragm reloading after extubation resulted in a complete reversal towards control levels of both proteases calpain and caspase-3 after 12 h of reloading and this was associated with normalization of diaphragm function [11]. By contrast, Thomas and colleagues did not observe any amelioration of protease activity after 4–7 h of diaphragmatic reloading [9], although animals in their study did most likely have less diaphragm reloading due to sedation and diaphragm fully recovered normal function. This suggests that the calpain system restoration is not only time dependent but also requires a certain amount of reloading to normalize. In addition, these observations also underline that the down regulation of these proteases is not required for diaphragm force to recover.

We could not detect changes in macrophagic or neutrophilic invasion between the groups in contrast to Thomas and colleagues. Possibly, different degrees of reloading cause subsequently different inflammatory responses.

### Weaning did not alter the decrease in protein synthesis

As one major upstream between gaining of proteins (protein synthesis) and proteolysis acts AKT. It increases protein synthesis in the phosphorylated state and, while dephosphorylated, AKT can induce the autophagy pathway via mammalian target of Rapamycin [28, 29]. In the current study, a decrease of pAKT/AKT was observed during mechanical ventilation, comparable to former studies [28]. This ratio remained reduced after both weaning periods, indicating that protein synthesis was still impaired with diaphragm reloading regardless of the load applied to the diaphragm. It is therefore unlikely that it contributed to the worsening of diaphragm function seen after weaning.

### Model validation

Gradual reloading is used to protect the muscle from overload-induced damage and to increase diaphragmatic reloading while reducing inspiratory pressure support [30]. We designed the PSV group closely to clinical needs while beginning with support-level identical to the inspiratory pressure during MV, gradual reduction, and adaption based on respiratory physiology [13]. By decreasing pressure support, we surely induced an increase in diaphragmatic activation. We re-increased the pressure level during PSV when the respiratory rate increased above 100 and/or hypercapnia occurred. It was impossible in this model due to the ventilator type to measure exact tidal volumes to calculate Vt/RR quotient or assess distress as this can be done in awake patients breathing through a tracheal cannula. Therefore our approach to guide the PSV setting was close to but not exactly mimicking the clinical situation during the weaning process. Nevertheless, even if the ventilator is set in the best clinical practice in patients undergoing pressure support ventilation, over-assistance in the clinical setting may be apparent [31]. Over-assistance may be most likely the reason for the worsening of diaphragm function in our study.

The frequency and duration of the spontaneous breathing trials was chosen closely to the work of Gayan-Ramirez and others, demonstrating preservation from VIDD even by these short terms of interruption from mechanical ventilation [8]. However, even in the beginning of a weaning process, short trials of max 5 min several times a day may be used to initiate weaning from the ventilator. It is important to notice that our approach to implement hourly spontaneous breathing intervals was based on scientific reasons by Gayan-Ramirez and colleagues demonstrating preservation of myogenic signalling using this strategy (see above) [8] and cannot mimic the clinical scenario of a usually 30 min lasting first spontaneous breathing trial that is prolonged in the following weaning attempts [32].

### Limitations of the model and clinical impact

We chose our established rat model to investigate the effects of different reloading approaches after MV, those are appropriate for the animal model but differ from those used in clinical practice. There is, however, evidence that the same pathways are activated in rats and humans and therefore findings in these animal models can be transferred to humans [33, 34]. However, our model is the first to describe biological effects of gradual vs. intermittent reloading on the diaphragm, which is not proven in humans, yet.

Over-assistance during PSV and due to the spontaneous breathing trial duration might have influenced these results. Hudson and colleagues revealed that over-assistance in pressure support ventilation cannot prevent from VIDD [35] and could not reproduce the findings of Futier and colleagues that demonstrated preservation of diaphragm metabolism by pressure support ventilation [36]. The actual load might therefore be essential for the effects on diaphragm function. Nevertheless, prevention, i.e. remaining sufficient load is different than regaining of force as in our model.

Additionally we have to address the influence of the sedative on the respiratory effort. Vaschetto and colleagues impressively demonstrated that the breathing effort during pressure support ventilation is dose-dependent of the sedative agent [37]. Our animals were sedated keeping them in a state of low sedation that suppressed moving and vegetative disturbance. However we could not monitor or titrate this sedation compared to the mentioned study [37].

We decided not to add another control group of 24 h of mechanical ventilation. Studying the rats under mechanical ventilation after 24 h of mechanical ventilation to address, whether weaning would improve the loss of diaphragm force caused by 12 h mechanical ventilation, may not be fully suitable. Indeed, after 12 h of mechanical ventilation, diaphragm force is reduced and we wanted to know which weaning approach would lead to more efficient restoration of diaphragm function, when weaning the rats at this time point.

Additionally, the weaning period of 12 h was chosen after the discrepant results from our study [11] and Thomas et al. [9] study, which have been described above. In our study investigating recovery with full diaphragm load [11], we have explained that the on-going decline in contractile force after 12 h of full load might have been due to overload that would be absent in a gradual reloading as the one chosen in the actual work. The decline in force despite loading is from our perspective due to insufficient loading.

Additionally protease activation, removal of oxidized proteins and restoration of function may also be influenced by time; reduction of protease action may be a first step to re-balance cellular homeostasis, but contractile force needs intact –replaced- sarcomeres to act. Therefore our results can only be interpreted in the investigated time frame of unloading and reloading.

Does this study help to understand what happens in weaning patients? At first, patients are weaned in more than 12 h and even severely ill individuals can get weaned. Importantly, our data indicate that the amount of load is decisive for weaning success.

## Conclusions

Pressure support ventilation with support levels that may replace diaphragmatic work completely is insufficient to regain force, and even short durations of SBT have the same effect. Continuous clinical assessment according to the guidelines of patients undergoing spontaneous breathing trials is therefore of high importance. The guidance by trans-diaphragmatic pressure monitoring or assessment of diaphragmatic electric activity might be able to guide weaning individually.

## Additional file

Additional file 1: Figure S1. Arterial blood gas data in the CMV, PSV and SBT groups. Values are expressed as means ± SD. (JPG 717 kb)

## Abbreviations

4HNE: Diaphragmatic 4-hydroxynonenal
AKT: Protein kinase B
ANOVA: One-way analysis of variance
CON: Acutely euthanized control group
MV: Mechanical ventilation
pAKT: Phosphorylated protein kinase B
PSV: Pressure support ventilation
ROS: Reactive oxygen species
SBT: Spontaneous breathing trial
SDS: Sodium dodecyl sulphate
VIDD: Ventilator-induced diaphragmatic dysfunction
