# Supplementary figures and images for: Influence of weaning methods on the diaphragm after mechanical ventilation in a rat model
Source: BMC Pulm Med. 2016 Aug 24;16(1):127. doi: 10.1186/s12890-016-0285-2 (PMC4997706; doi:10.1186/s12890-016-0285-2)

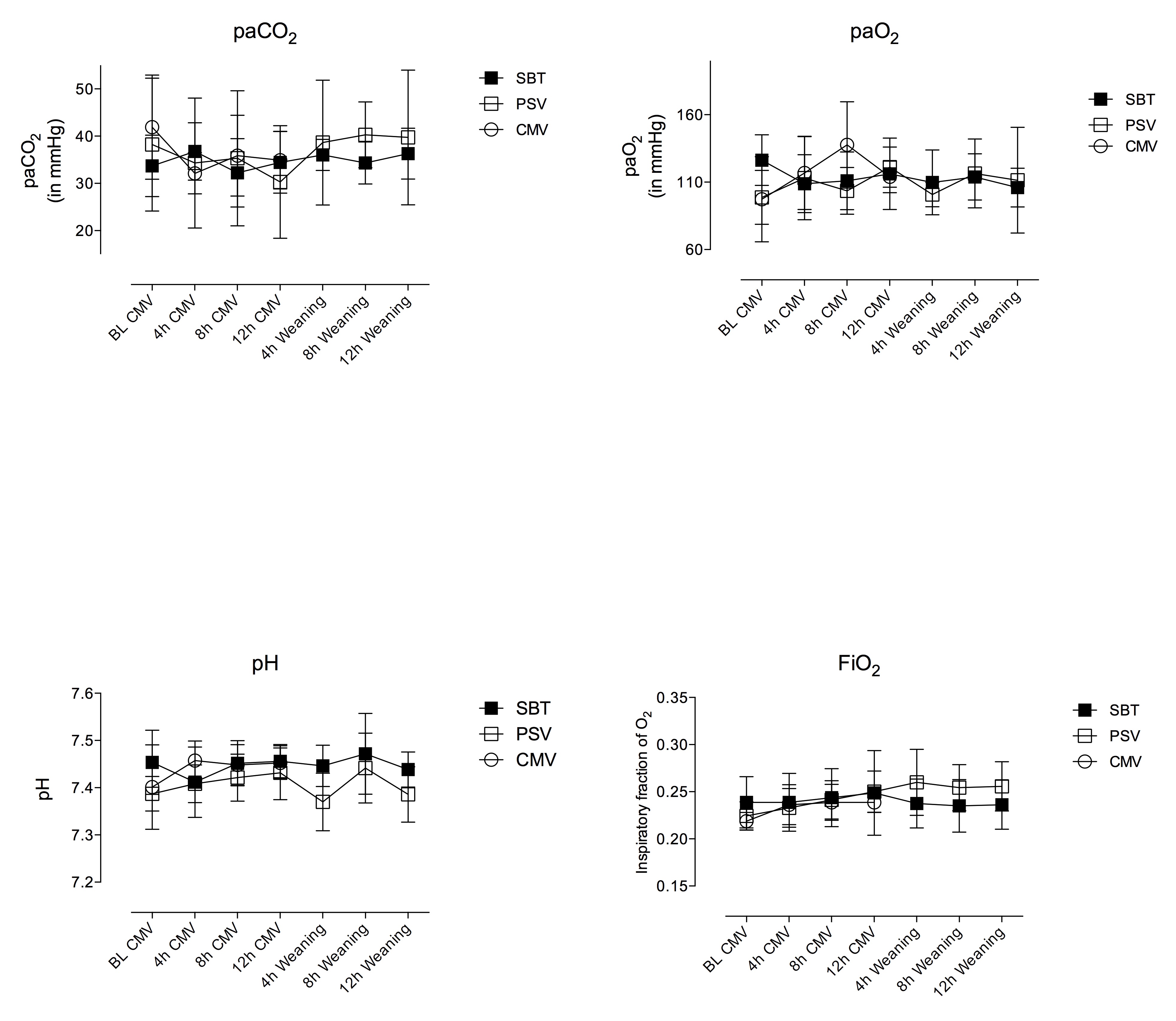

Supplement: Additional file 1: Figure S1. — Arterial blood gas data in the CMV, PSV and SBT groups. Values are expressed as means ± SD. (JPG 717 kb) [file 12890_2016_285_MOESM1_ESM.jpg]
